# Association of obesity profiles with type 2 diabetes in Chinese adults: Findings from the China health and nutrition survey

**DOI:** 10.3389/fnut.2022.922824

**Published:** 2022-09-13

**Authors:** Siting Zhang, Weiyi Li, Xiaofang Jia, Jiguo Zhang, Hongru Jiang, Liusen Wang, Huijun Wang, Bing Zhang, Zhihong Wang, Gangqiang Ding

**Affiliations:** National Institute for Nutrition and Health, Chinese Center for Disease Control and Prevention, Beijing, China

**Keywords:** obesity, body mass index, waist circumference, type 2 diabetes, China

## Abstract

**Aims:**

To examine longitudinal associations of obesity profiles, continuous BMI, and waist circumference (WC) with the risk of type 2 diabetes in Chinese adults.

**Methods:**

Data were derived from three waves (2009, 2015, and 2018) of the China Health and Nutrition Survey, and 3,595 adults aged 18–65 years who participated in at least two waves of the survey and had completed data were analyzed. Obesity profiles included BMI- or WC-related single obesity and combined obesity. Combined obesity was categorized into six groups including Group 1 with normal BMI and WC, Group 2 with normal BMI but pre-abdominal obesity, Group 3 with normal BMI but abdominal obesity, Group 4 with abnormal BMI (overweight and general obesity) and normal WC, Group 5 with abnormal BMI and pre-abdominal obesity, and Group 6 with abnormal BMI and abdominal obesity. Three-level mixed-effects logistic regressions with random intercept stratified by gender and restricted cubic splines were performed to examine ORs and 95%CIs for the risk of type 2 diabetes.

**Results:**

In men, compared with subjects of Group 1, those in Group 3 had higher risk, with an OR of 4.83 (95% CI: 1.99–11.74), followed by those in Group 6 (OR = 4.05, 95%CI: 2.32–7.08) and Group 5 (OR = 2.98, 95%CI: 1.51–5.87) after adjusting for all potential confounders. For women, the subject of Group 6 had highest risk (OR = 8.79, 95%CI: 4.04–19.12), followed by Group 3 (OR = 3.30, 95%CI: 1.23–8.86) and Group 5 (OR = 3.16, 95%CI: 1.21–8.26). No significant association between abnormal BMI and normal WC (Group 4) was observed in both genders. Type 2 diabetes risk increased steeply at BMI of 23.5 kg/m^2^ and 22.5 kg/m^2^ or higher, and WC of 82.0 cm and 83.0 cm or higher in Chinese adult men and women, respectively (*p* for overall <0.001).

**Conclusion:**

Chinese adults with pre-abdominal or abdominal obesity had a relative high risk of type 2 diabetes independent of BMI levels. Lower BMI (≤23.5 kg/m^2^ for men and ≤22.5 kg/m^2^ for women) and lower WC (82.0 cm for men and ≤83.0 cm for women) values than the current Chinese obesity cut-offs were found to predict the risk of type 2 diabetes. These findings urge to inform WC modification and optimization of early screening guidelines.

## Introduction

Diabetes has been one of the fastest growing health issues reaching alarming levels since the 21st century, an estimated 537 million (standardized prevalence: 9.8%) adults aged 20–79 years are living with diabetes around the world in 2021 (1), and type 2 diabetes accounts for more than 90% of all patients with diabetes (2). The estimated prevalence of diabetes increased steadily from 10.9% in 2013 to 12.4% in 2018 among Chinese adults (3), and the severe disease situation may lead to more cardiovascular complications that put a huge burden on patients, caregivers, and healthcare systems. Thus, it is significant to explore the modifiable factors for type 2 diabetes prevention and management.

Although the risk factors and causes of type 2 diabetes have not been completely ascertained, there were strong positive associations with overweight, general obesity, abdominal obesity, physical activities, lifestyle, and family history (4, 5). Among these factors, the causal effect of obesity on the risk of type 2 diabetes and insulin resistance had been identified by a two-sample mendelian randomization study (6), and a large proportion of type 2 diabetes incident cases could be attributed to excess weight (PARs: women:48.6%; men:41.5%) and abdominal obesity (PARs: women:50.4%; men: 30.3%) in Chinese adults (7). Several studies also indicated that the Asian population had a relatively higher body fat percentage which predisposed them to prediabetes and diabetes at the same BMI compared to other ethnic groups (8, 9). BMI and waist circumference (WC) deserve comprehensive consideration to study whether obesity might play a superimposed role in type 2 diabetes.

Currently, several studies have shown that the association of obesity with type 2 diabetes varied across different obesity types. For example, abdominal obesity was found to be more predictive for incident diabetes than general obesity (10). Lu et al. also cross-sectionally reported that abdominal (OR = 1.55, 95%CI: 1.08–2.24) and compound obesity (OR = 1.85, 95%CI: 1.25–2.73) were highly associated with the risk of type 2 diabetes development (11). Previous studies had indicated that a composite of BMI and waist circumference (WC) might be a better obesity metric for risk of type 2 diabetes (12). However, to our knowledge, few studies focused on the remarkable effects of obesity across different BMI and WC combinations on type 2 diabetes, just highlighted the impact of special types which could not cover the obese completely. Moreover, the early warning and screening cut-offs of BMI and WC for obesity based on type 2 diabetes are still controversial in China (13, 14). It is, therefore, of importance to identify the associations of different obesity types and their anthropometric markers with the risk of type 2 diabetes.

In this study, we investigated the odds ratios of combined obesity across BMI- and WC-related single obesity groups, and exposure–response relationships between BMI/WC and type 2 diabetes risk using the data of Chinese adults aged 18–65 from the China Health and Nutrition Survey (CHNS).

## Materials and methods

### Study population

Data were derived from the China Health and Nutrition Survey (CHNS), an ongoing longitudinal study conducted in 15 provinces (autonomous regions, municipalities) of China. The CHNS is committed to evaluating the impacts of sociological, economic, and demographic changes in numerous nutrition- and health-related outcomes of the Chinses population. Eleven consecutive rounds of follow-up surveys had been carried out from 1989 to 2018, and multistage, random cluster sampling was used to draw the survey sample from each province to ensure representativeness in the CHNS. In each province, the counties and cities were stratified by income, and a weighted sampling scheme was used to select two cities and four counties. Villages and townships within the counties and urban and suburban neighborhoods within the cities were randomly selected. The communities were selected randomly as the primary sampling units, and in each community, 20 households were randomly selected, and all household members were interviewed. Such sampling reflects the hierarchical data structure of the CHNS: measurement occasions (level 1) for individuals (level 2) nested in communities (level 3). More details regarding the survey design and methods have been described in a previous report (15).

This study used data from three waves of CHNS conducted in 2009, 2015, and 2018 because fasting vein blood was only collected in these rounds. Subjects aged 18 to 65 who participated in at least two waves of surveys and had completed data on socio-economy, demography, anthropometry, biochemical measurements, dietary intake, and other lifestyle factors were included. We exclude pregnant or lactating women (n = 208 responses), those with a BMI <18.5 kg/m^2^ (*n* = 553 responses), implausible energy intakes (n = 104 responses; for men consuming <800 kcal per day or >6,000 kcal, for women consuming < 600 kcal or >5,000 kcal), those having baseline diabetes (*n* = 1,109 subjects). The final analytical sample, therefore, included 3,595 participants clustered in 268 communities, resulting in 8,347 total responses (2,280, 3,283, and 2,784 responses in 2009, 2015, and 2018, respectively). The number of participants who responded two times and three times was 2,438 and 1,157.

All participants gave written informed consent, and the survey protocol was approved by the Institutional Review Committees of the University of North Carolina at Chapel Hill and the Chinese Center for Disease Control and Prevention(No. 201524).

### Assessment of obesity profiles

Trained health workers or nurses measured height, weight, and WC following the standardized procedures. According to validated anthropometry manual standards (16), weight and height were measured to the nearest 0.1 kg and 0.1 cm, respectively, with the participants in lightweight clothing and without shoes. BMI was calculated as weight (kg) divided by the square of height (m^2^). WC was measured with an inelastic flexible ruler to the nearest 0.1 cm, circled around the horizontal position of the midpoint between the lower margin of the rib and the attachment of the iliac crest.

Obesity profiles included BMI- or WC-related single obesity and combined obesity. Based on the criteria of weight for adults in China (17), general obesity was defined as BMI ≥ 28.0 kg/m^2^, overweight as 24.0 ≤ BMI < 28.0 kg/m^2^, and normal BMI as 18.5 ≤ BMI < 24.0 kg/m^2^. Abdominal obesity was defined as WC ≥ 90 cm for men and ≥ 85 cm for women, pre-abdominal obesity as 85 ≤ WC < 90 cm for men and 80 ≤ WC < 85 cm for women, and normal WC as WC < 85 cm for men and < 80 cm for women. Therefore, single BMI-related groups consisted of abnormal BMI (overweight and general obesity) and normal BMI, and single WC-related groups consisted of abdominal obesity, pre-abdominal obesity, and normal WC in our study.

Based on the above criteria, combined obesity was reclassified across BMI- and WC-related single groups by gender, respectively. Group 1 represents normal BMI and WC, Group 2 represents normal BMI and pre-abdominal obesity (P-AO), Group 3 represents normal BMI and abdominal obesity (AO), Group 4 represents abnormal BMI (general obesity and overweight) with normal WC, Group 5 represents abnormal BMI and obesity-AO, and Group 6 represents abnormal BMI and AO.

### Diagnosis of type 2 diabetes

Fasting blood samples were collected by trained physicians or nurses *via* venipuncture, and centrifugations were performed within 3 h in order to obtain reliable test results. Blood samples were preserved at −2~8°C for short-term storage and later laboratory analysis. Detection of fasting blood glucose (FPG) and glycated hemoglobin A_1c_ (HbA_1c_) used GOD-PAP and HLC/HLC/HPLC methods by determiner regents on the Hitachi 7600 (Randox, UK) and HLC-723 G7/D10/PDQ A1c (Tosoh, Japan/ Bio-Rad, USA/ Primus, USA) automated analyzers, respectively.

According to ADA criteria (18), type 2 diabetes was defined as having at least one of the following: (a) FPG ≥ 7.0 mmol/L, (b) HbA_1c_ ≥ 48.0 mmol/mol (6.5%), and (c) having treatments for diabetes including oral hypoglycemic medication or insulin injections.

### Assessment of covariates

Trained investigators used standard questionnaires to collect information on socio-demographic and lifestyle variables, including gender; age (in years); per capita annual family income (tertile); education level (primary school and below, completed middle school, high school, and above); geographic region (rural and urban); community urbanization index (score) (19); smoking (current smokers vs. former or non-smokers); alcohol drinking (current drinkers vs. former or non-drinkers); sleep duration (6–9 vs. <6 or >9 h) (20); physical activity (in MET hours/week) (21). In addition, we also assessed other potential dietary confounders, including total energy intake (TEI), energy supply ratio of carbohydrate and protein, intakes of dietary fiber, calcium, Vitamin C, and retinol calculated from data collected by consecutive 3 days 24 h recalls combined with the weighing of household seasonings.

### Statistical analysis

First, statistical interaction tests between combined obesity and gender were performed, and a significant interaction was founded. For the baseline characteristics of the participants, mean and SDs values were used to present the distribution of continuous variables, percentage of the total for categorical variables was expressed, and differences among diverse groups were examined using the Kruskal–Wallis H test for continuous variables and Chi-square test for categorical variables. In view of the hierarchical data structure of the CHNS, we performed a likelihood ratio test to choose the preferred multilevel mixed-effects logistic regression model and found that the three-level mixed-effects logistic regression model with random intercept was able to estimate the odds ratios (ORs) of type 2 diabetes well, which taken communities as level 3, individuals as level 2, and repeated measurement occasions for individuals as level 1. In this regression model, combined obesity, different levels of BMI and WC were fixed effects, and community and individual were random effects. The intra-class correlation (ICC) was calculated to compare the variability within communities and among individuals. Three models were constructed for analysis: Model 1 adjusted for no covariates. Model 2 adjusted for age, income, education, urbanicity index, geographic region, smoking, alcohol drinking, sleep duration, and physical activity. Model 3 additionally adjusted for intakes of total energy, fiber, and other related dietary factors.

The currently used obesity cut-offs for screening high-risk groups of diabetes were overweight for BMI and abdominal obesity for WC among Chinese adults. To fully examine the potential dose–response relationship between BMI and type 2 diabetes, we divided the participants into five groups (18.5, 23.0, 25.0, 27.0 kg/m^2^~, and ≥ 30 kg/m^2^), based on relevant BMI cut-off points recommended by WHO and Asian criteria (22, 23). Linear trends were evaluated across increasing categories of BMI and WC by assigning participants the median values to levels of their BMI and WC and modeling this variable as a continuous term. Furthermore, the possible exposure–response relationships between continuous BMI and WC and the risk of type 2 diabetes were explored, using a restricted cubic spline function with four knots (located at the fifth, 35th, 65th, and 95th percentiles). According to the Guideline for the prevention and treatment of type 2 diabetes in China (2020 edition), the Chinese Diabetes Risk Score included age, sex, family history of diabetes, systolic blood pressure, BMI, and waist circumference, and different levels of these indicators were assigned different scores (24). As shown in Supplementary Table 1, our study chose BMI and WC levels with a diabetes risk score of 0 as the reference group for all spline plots (22.0 kg/m^2^ for BMI; 75 cm and 70 cm of WC for men and women, respectively).

All statistical analyses were conducted in SAS 9.4 (SAS Institute Inc., Cary, NC, USA) and Stata 15SE (Stata Corp., College Station, TX, USA). All statistical tests were two-sided and considered significant at *p* < 0.05.

## Results

### Baseline characteristics

Among the 3595 participants, the median follow-up time was 6 years, ranging from 3 to 9 years. The total person-years and cumulative number of cases of type 2 diabetes were 22,294 and 349. There are 1,585 men and 2010 women at baseline in this study, and their mean age was 45.4 years. The selected characteristics of participants across six groups are summarized by gender in Table 1. In men and women, the proportions of abnormal BMI or WC were 58.6% and 66.8% (Groups 2–6), the proportions of abnormal BMI (general obesity/overweight) were 49.1 and 47.9% (Group 4,5,6), and the proportions of abnormal WC (pre-abdominal/abdominal obesity) were 48.6 and 61.9% (Group 2, 3, 5, 6), respectively. Of all participants, the subtypes for age group, per capita annual family income levels, smoking status, alcohol drinking status, BMI, WC, FPG, and intakes of fiber were statistically significant (*p* < 0.05).

**Table 1:** Baseline characteristics of adults across obesity profiles stratified by gender, CHNS^†^.

**Baseline Characteristics**	**Men**						**Women**					
	**Normal**	**Normal**	**Normal**	**Abnormal**	**Abnormal**	**Abnormal**	**Normal**	**Normal**	**Normal**	**Abnormal**	**Abnormal**	**Abnormal**
	**BMI**	**BMI**	**BMI**	**BMI**	**BMI**	**BMI**	**BMI**	**BMI**	**BMI**	**BMI**	**BMI**	**BMI**
	**and WC**	**and P-AO**	**and AO**	**and normal**	**and P-AO**	**and AO**	**and WC**	**and P-AO**	**and AO**	**and normal**	**and P-AO**	**and AO**
				**WC**						**WC**		
	**(Group 1)**	**(Group 2)**	**(Group 3)**	**(Group 4)**	**(Group 5)**	**(Group 6)**	**(Group 1)**	**(Group 2)**	**(Group 3)**	**(Group 4)**	**(Group 5)**	**(Group 6)**
	***n* = 656**	***n* = 109**	***n* = 42**	***n* = 159**	***n* = 185**	***n* = 434**	***n* = 667**	***n* = 230**	***n* = 150**	***n* = 99**	***n* = 204**	***n* = 660**
Age (years)	44.17 ± 10.09	47.83 ± 8.75	46.78 ± 7.66	44.87 ± 8.78	46.25 ± 8.75	46.48 ± 9.19*	42.99 ± 9.13	46.28 ± 9.31	47.54 ± 8.37	43.52 ± 8.08	45.83 ± 8.02	47.29 ± 8.34*
Income level (%)												
Low	320 (48.34)	43 (6.50)	20 (3.02)	63 (9.52)	60 (9.06)	156 (23.56)*	335 (37.06)	107 (11.84)	59 (6.53)	46 (5.09)	88 (9.73)	269 (29.76)*
Medium	221 (37.39)	42 (7.11)	16 (2.71)	63 (10.66)	74 (12.52)	175 (29.61)	216 (30.25)	75 (10.50)	55 (7.70)	42 (5.88)	70 (9.80)	256 (35.85)
High	115 (34.64)	24 (7.23)	6 (1.81)	33 (9.94)	51 (15.36)	103 (31.02)	116 (29.59)	48 (12.24)	36 (9.18)	11 (2.81)	46 (11.73)	135 (34.44)
Education level (%)												
Primary school and below	144 (48.16)	24 (8.03)	8 (2.68)	24 (8.03)	28 (9.36)	71 (23.75)	184 (29.21)	67 (10.63)	45 (7.14)	36 (5.71)	72 (11.43)	226 (35.87)
Middle school	295 (40.97)	46 (6.39)	21 (2.92)	74 (10.28)	85 (11.81)	199 (27.64)	278 (35.37)	87 (11.07)	63 (8.02)	30 (3.82)	78 (9.92)	250 (31.81)
High school and above	217 (38.34)	39 (6.89)	13 (2.30)	61 (10.78)	72 (12.72)	164 (28.98)	205 (34.51)	76 (12.79)	42 (7.07)	33 (5.56)	54 (9.09)	184 (30.98)
Geographic region (%)												
Rural	459 (41.58)	74 (6.70)	29 (2.63)	110 (9.96)	115 (10.42)	317 (28.71)	466 (33.65)	146 (10.54)	92 (6.64)	66 (4.77)	145 (10.47)	470 (33.94)
Urban	197 (40.96)	35 (7.28)	13 (2.70)	49 (10.19)	70 (14.55)	117 (24.32)	201 (32.16)	84 (13.44)	58 (9.28)	33 (5.28)	59 (9.44)	190 (30.40)
Urbanicity index	68.99 ± 16.73	72.54 ± 17.21	70.72 ± 15.64	72.74 ± 15.94	74.48 ± 15.92	71.19 ± 16.72*	71.68 ± 16.97	71.42 ± 17.59	71.99 ± 17.64	69.51 ± 16.84	70.70 ± 16.49	70.29 ± 16.23
Smoking (%)												
Former/Non-smoker	368 (41.21)	43 (4.82)	13 (1.46)	108 (12.09)	123 (13.77)	238 (26.65)*	627 (34.51)	204 (11.23)	122 (6.71)	96 (5.28)	193 (10.62)	575 (31.65)*
Current smoker	288 (41.62)	66 (9.54)	29 (4.19)	51 (7.37)	62 (8.96)	196 (28.32)	40 (20.73)	26 (13.47)	28 (14.51)	3 (1.55)	11 (5.70)	85 (44.04)
Alcohol drinking (%)												
Former/Non-drinker	319 (42.82)	32 (4.30)	14 (1.88)	96 (12.89)	95 (12.75)	189 (25.37)*	590 (35.10)	187 (11.12)	114 (6.78)	91 (5.41)	179 (10.65)	520 (30.93)*
Current drinker	337 (40.12)	77 (9.17)	28 (3.33)	63 (7.50)	90 (10.71)	245 (29.17)	77 (23.40)	43 (13.07)	36 (10.94)	8 (2.43)	25 (7.60)	140 (42.55)
Sleep duration (%)												
6~9 h	537 (40.59)	94 (7.11)	38 (2.87)	140 (10.58)	157 (11.87)	357 (26.98)	574 (34.11)	192 (11.41)	116 (6.89)	87 (5.17)	169 (10.04)	545 (32.38)
<6/>9 h	119 (45.42)	15 (5.73)	4 (1.53)	19 (7.25)	28 (10.69)	77 (29.39)	93 (28.44)	38 (11.62)	34 (10.40)	12 (3.67)	35 (10.70)	115 (35.17)
Physical activity (MET hours/week)	221.85 ± 185.24	230.40 ± 202.00	189.41 ± 145.57	204.69 ± 188.11	204.88 ± 195.47	204.25 ± 176.54	226.19 ± 199.69	209.10 ± 196.84	201.21 ± 177.55	226.42 ± 187.25	216.34 ± 184.46	216.36 ± 214.59
BMI (kg/m^2^)	21.40 ± 1.40	22.55 ± 1.08	22.59 ± 1.14	25.40 ± 1.25	25.77 ± 1.58	27.88 ± 2.65*	21.35 ± 1.42	22.38 ± 1.16	22.50 ± 1.15	25.38 ± 1.52	25.61 ± 1.57	27.49 ± 2.40*
WC (cm)	76.10 ± 5.72	86.72 ± 1.42	92.64 ± 2.86	79.98 ± 5.05	87.02 ± 1.48	96.65 ± 5.62*	73.06 ± 4.35	81.68 ± 1.49	88.59 ± 3.77	75.93 ± 3.62	82.14 ± 1.54	92.78 ± 6.08*
FPG (mmol/L)	5.00 ± 0.55	5.22 ± 0.66	5.24 ± 0.62	5.15 ± 0.54	5.25 ± 0.64	5.30 ± 0.61*	5.01 ± 0.53	5.07 ± 0.62	5.04 ± 0.58	5.01 ± 0.49	5.14 ± 0.53	5.23 ± 0.56*
Dietary intake												
Total energy (kcal/d)	2,319.55 ± 735.07	2,443.80 ± 789.75	2,274.91 ± 867.52	2,284.91 ± 775.21	2,272.36 ± 818.26	2,337.39 ± 723.23	2,021.04 ± 604.53	2,044.39 ± 666.67	2,182.44 ± 690.38	2,065.70 ± 582.30	2,006.97 ± 604.67	2,046.27 ± 631.09
Carbohydrate (% of energy)	52.37 ± 12.11	50.12 ± 11.79	48.62 ± 14.87	51.38 ± 12.22	50.65 ± 11.39	52.28 ± 11.88	52.83 ± 11.46	52.63 ± 12.36	51.44 ± 11.73	53.28 ± 11.96	52.84 ± 11.44	53.08 ± 11.57
Protein (% of energy)	12.34 ± 2.82	12.48 ± 2.88	12.86 ± 2.63	12.51 ± 2.91	12.89 ± 3.01	12.67 ± 2.75	12.77 ± 3.19	12.73 ± 2.95	12.74 ± 3.05	12.05 ± 2.68	12.62 ± 2.99	12.69 ± 2.65
Fiber (g/d)	12.10 ± 7.71	12.52 ± 8.30	11.88 ± 7.60	12.60 ± 10.32	13.06 ± 12.39	12.84 ± 6.89*	11.47 ± 6.55	12.41 ± 15.76	12.45 ± 5.93	12.20 ± 6.32	11.39 ± 8.29	12.25 ± 7.13*
Calcium (mg/d)	391.63 ± 197.79	435.74 ± 257.74	448.25 ± 245.24	408.35 ± 260.06	435.44 ± 255.27	418.03 ± 323.19	381.55 ± 256.19	353.10 ± 153.58	381.20 ± 170.33	350.49 ± 166.31	338.37 ± 159.28	375.16 ± 248.98
Vitamin C (mg/d)	81.14 ± 102.21	80.85 ± 55.59	86.25 ± 60.56	73.40 ± 48.99	83.23 ± 53.24	93.75 ± 180.17	88.69 ± 152.96	79.46 ± 93.79	87.17 ± 73.78	78.44 ± 47.21	75.92 ± 49.72	86.45 ± 119.48
Retinol (μgRAE/d)	678.86 ± 1,025.44	589.58 ± 623.77	692.33 ± 687.28	754.65 ± 1,531.10	838.65 ± 1,415.44	583.50 ± 890.26	714.90 ± 943.92	541.34 ± 464.28	569.25 ± 699.57	510.55 ± 418.03	589.07 ± 1,031.21	587.53 ± 1,007.54*

† : Data are mean ± SD for continuous variables or n (%) for categorical variables.

* p < 0.05, p-values were calculated with Kruskal–Wallis H test and χ^2^ test for continuous and categorical variables, respectively. P-AO, pre-abdominal obesity; AO, abdominal obesity.

### Associations of different levels of BMI and WC with type 2 diabetes in participants

Tables 2, 3 show the ORs according to different levels of BMI and WC in Chinese adults stratified by gender, respectively. In men and women, the ICCs were 4.33 and 4.83% for BMI and 4.27 and 5.80% for WC, respectively, at the community level. It was suggested that the community level had a certain influence on the risk of type 2 diabetes. In both men and women, with the increasing levels of BMI and WC, the risk also significantly increased (*p*-trends < 0.001). In model 3 adjusted for all potential confounders, participants showed 4.37 times and 18.42 times higher risk of type 2 diabetes when BMI reached 30.0 kg/m^2^ in men and women, respectively, as compared with BMI level of 18.5– < 23.0 kg/m^2^ (men: OR = 4.37, 95% CI: 2.07–9.22; women: OR = 18.42, 95% CI: 7.58–44.72). In addition, compared with WC level of <75 cm, the ORs had been 4.56 (95% CI: 1.61–12.93) and 4.59 (95% CI: 1.68–12.52) for model 3 in men and women, respectively, when WC reached abdominal obesity stage (men: ≥ 90.0 cm; women ≥ 85.0 cm). And the ORs further went up to 9.15 (95%CI: 3.30–25.42) and 15.43 (95%CI: 5.36–44.43) when WC was >95 cm in men and women, respectively.

**Table 2:** Association between BMI levels and risk of type 2 diabetes among Chinese adults aged 18–65 in three-level mixed-effects logistic regression^†^.

	**Men**			**Women**		
	**Model 1**	**Model 2**	**Model 3**	**Model 1**	**Model 2**	**Model 3**
**Fixed effect**	Odds ratio (95% CI)					
BMI (kg/m^2^)						
18.5~	1	1	1	1	1	1
23.0~	2.01 (1.20, 3.36)*	1.91 (1.08, 3.36)*	1.93 (1.09, 3.42)*	3.04 (1.73, 5.36)*	3.05 (1.56, 5.95)*	3.28 (1.64, 6.56)*
25.0~	2.66 (1.60, 4.44)*	2.49 (1.41, 4.41)*	2.55 (1.44, 4.54)*	3.34 (1.87, 5.97)*	3.48 (1.74, 6.94)*	3.74 (1.82, 7.70)*
27.0~	3.12 (1.85, 5.29)*	2.84 (1.58, 5.10)*	2.78 (1.54, 5.03)*	4.73 (2.61, 8.57)*	4.86 (2.35, 10.06)*	5.15 (2.41, 10.97)*
>30.0	4.40 (2.30, 8.41)*	4.51 (2.15, 9.46)*	4.37 (2.07, 9.22)*	12.74 (6.73, 24.12)*	15.65 (6.78, 36.16)*	18.42 (7.58, 44.72)*
*p*-trend^‡^	<0.001	<0.001	<0.001	<0.001	<0.001	<0.001
**Random effect**	Covariance estimates (SE)					
Community	0.20 (0.17)	0.24 (0.22)	0.24 (0.23)	0.34 (0.21)	0.36 (0.29)	0.34 (0.30)
Individual	1.05 (0.58)	1.95 (0.75)	2.01 (0.99)	1.19 (0.63)	3.10 (1.44)	3.43 (1.55)
**Intra-class correlation (ICC, %)**
Community	4.38	4.38	4.33	7.11	5.38	4.83
Individual	27.54	39.94	40.64	31.84	51.30	53.40

† Model 1 adjusted for no covariates. Model 2 adjusted for age, income level, education level, urbanized index, geographic region, smoking, alcohol drinking, physical activity, and sleep duration. Model 3 additionally adjusted for total energy intake, energy supply ratio of carbohydrate and protein, fiber, calcium, vitamin C, and retinol. SE: standards of error of means.

‡ p-trend was calculated by assigning median values to each BMI group, and this variable was entered as a continuous term in the regression models.

* p < 0.05.

**Table 3:** Association between WC levels and risk of type 2 diabetes among Chinese adults aged 18–65, in three-level mixed-effects logistic regression^†^.

	**Men**			**Women**		
	**Model 1**	**Model 2**	**Model 3**	**Model 1**	**Model 2**	**Model 3**
**Fixed effect**	Odds ratio (95% CI)					
WC (cm)						
<75.0	1	1	1	1	1	1
75.0~	2.18 (0.73, 6.50)	1.88 (0.60, 5.92)	1.74 (0.55, 5.49)	1.65 (0.62, 4.41)	1.35 (0.46, 4.01)	1.39 (0.46, 4.24)
80.0~	3.35 (1.23, 9.14)*	2.90 (1.01, 8.29)*	2.79 (0.97, 8.01)	3.71 (1.56, 8.81)*	2.87 (1.09, 7.56)*	3.14 (1.15, 8.52)*
85.0~	4.63 (1.74, 12.34)*	3.80 (1.36, 10.65)*	3.81 (1.36, 10.68)*	5.61 (2.38, 13.23)*	4.35 (1.64, 11.48)*	4.59 (1.68, 12.52)*
90.0~	6.12 (2.28, 16.40)*	4.82 (1.70, 13.64)*	4.56 (1.61, 12.93)*	8.25 (3.45, 19.72)*	6.27 (2.31, 17.02)*	6.89 (2.44, 19.40)*
>95	11.29 (4.32, 29.47)*	9.62 (3.46, 26.75)*	9.15 (3.30, 25.42)*	15.41 (6.59, 36.07)*	12.99 (4.73, 35.65)*	15.43 (5.36, 44.43)*
*p*-trend^‡^	<0.001	<0.001	<0.001	<0.001	<0.001	<0.001
**Random effect**	Covariance estimates (SE)					
Community	0.21 (0.17)	0.22 (0.21)	0.22 (0.21)	0.40 (0.22)	0.44 (0.31)	0.42 (0.32)
Individual	0.95 (0.58)	1.71 (0.90)	1.73 (0.93)	1.18 (0.63)	3.23 (1.52)	3.54 (1.63)
**Intra-class correlation (ICC, %)**
Community	4.77	4.13	4.27	8.13	6.36	5.80
Individual	26.08	36.95	37.29	32.36	52.78	54.61

† Model 1 adjusted for no covariates. Model 2 adjusted for age, income level, education level, urbanized index, geographic region, smoking, alcohol drinking, physical activity, and sleep duration. Model 3 additionally adjusted for total energy intake, energy supply ratio of carbohydrate and protein, fiber, calcium, vitamin C, and retinol. SE: standards of error of means.

‡ p-trend was calculated by assigning median values to each WC group, and this variable was entered as a continuous term in the regression models.

* p < 0.05.

### Exposure–response relationships between continuous BMI/ WC and type 2 diabetes in participants

**Figure 1:**
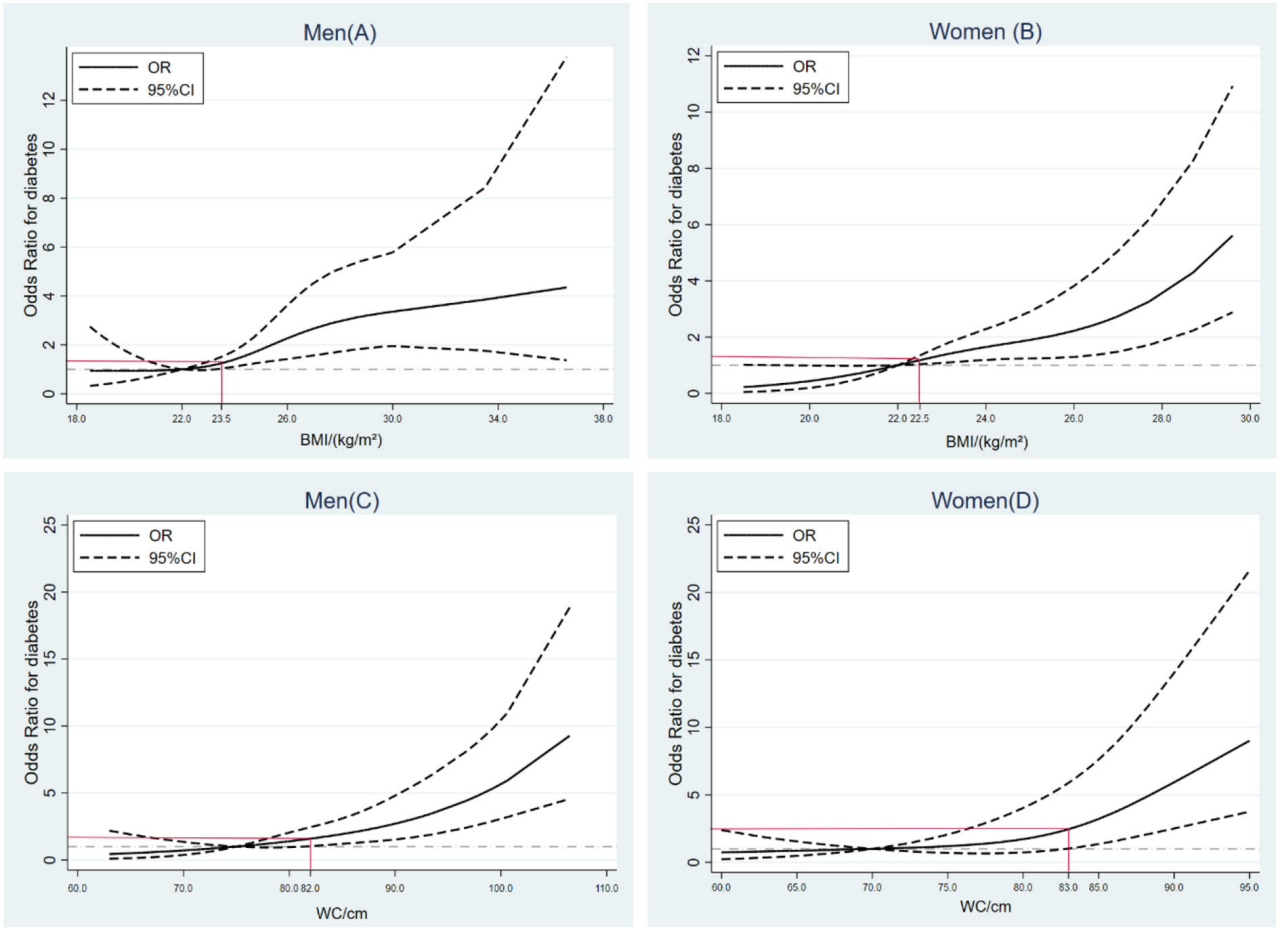
Exposure–response relationships between BMI/waist circumference (WC) and risk of type 2 diabetes by restricted cubic spline for adjusted modified logistic regression model with the estimation of odds ratio.

As shown in Figure 1, the linear relationship between continuous BMI and WC and the risk of Type 2 diabetes were observed adjustment for all potential confounders (*p* for non-linear > 0.05). Taking 22.0 kg/m^2^ as a BMI reference, the ORs (95% CI) were 1.25 (1.03, 1.51) at 23.5 kg/m^2^ for men, and 1.18 (1.03, 1.35), at 22.5 kg/m^2^ for women. Similarly, taking 75 cm and 70 cm as WC references for men and women, respectively, the ORs (95% CI) were 1.59 (1.03, 2.45) at 82.0 cm for men, and 2.46 (1.02, 5.89) at 83.0 cm for women.

### Associations of obesity profiles with type 2 diabetes in participants

Table 4 shows the longitudinal association between different groups and the odds ratio (95%CI) of type 2 diabetes, respectively, in Chinese adults stratified by gender. After adjusting for potential confounders in each model, in men, compared with Group 1, those in Group 3 with normal BMI and AO showed significantly highest ORs, followed by Group 6 with abnormal BMI and AO, and Group 5 with abnormal BMI and P-AO, For women, those in Group 6 with abnormal BMI and AO were related to higher risk of type 2 diabetes, followed by Group 2 and Group 3 with normal BMI but P-AO/AO.

**Table 4:** Association between combined obesity and risk of type 2 diabetes among Chinese adults aged 18–65, in three-level mixed-effects logistic regression^†^.

	**Men**			**Women**		
	**Model 1**	**Model 2**	**Model 3**	**Model 1**	**Model 2**	**Model 3**
**Fixed effect**	Odds ratio (95% CI)					
Normal BMI and WC (Group 1)	1	1	1	1	1	1
Normal BMI and P-AO (Group 2)	1.53 (0.66, 3.57)	1.27 (0.51, 3.14)	1.30 (0.52, 3.23)	3.63 (1.66, 7.92)*	2.74 (1.13, 6.62)*	2.90 (1.16, 7.23)*
Normal BMI and AO (Group 3)	5.35 (2.43, 11.77)*	4.93 (2.05, 11.88)*	4.83 (1.99, 11.74)*	4.50 (1.96, 10.33)*	3.13 (1.21, 8.11)*	3.30 (1.23, 8.86)*
Abnormal BMI and normal WC (Group 4)	1.95 (0.95, 4.00)	1.88 (0.86, 4.10)	1.82 (0.83, 3.99)	1.98 (0.67, 5.88)	2.02 (0.60, 6.78)	1.99 (0.57, 6.94)
Abnormal BMI and P-AO (Group 5)	2.97 (1.61, 5.47)*	2.85 (1.46, 5.59)*	2.98 (1.51, 5.87)*	2.93 (1.29, 6.64)*	2.91 (1.15, 7.34)*	3.16 (1.21, 8.26)*
Abnormal BMI and AO(Group 6)	4.54 (2.77, 7.42)*	4.13 (2.37, 7.17)*	4.05 (2.32, 7.08)*	8.76 (4.70, 16.31)*	7.98 (3.82, 16.69)*	8.79 (4.04, 19.12)*
**Random effect**	Covariance estimates (SE)					
Community	0.21 (0.17)	0.23 (0.22)	0.24 (0.23)	0.33 (0.21)	0.33 (0.28)	0.30 (0.29)
Individual	1.09 (0.61)	1.94 (0.99)	1.97 (1.03)	1.26 (0.63)	3.23 (1.47)	3.60 (1.62)
**Intra-class correlation (ICC, %)**						
Community	4.48	4.24	4.42	6.78	4.85	4.22
Individual	28.29	39.74	40.22	32.60	51.98	54.26

† : Model 1 adjusted for no covariates. Model 2 adjusted for age, income level, education level, urbanized index, geographic region, smoking, alcohol drinking, physical activity, and sleep duration. Model 3 additionally adjusted for total energy intake, energy supply ratio of carbohydrate and protein, fiber, calcium, vitamin C, and retinol. P-AO, pre-abdominal obesity; AO, abdominal obesity; SE, standards of error of means.

* p < 0.05.

After adjustment for all covariates, the ICCs were 4.42% and 4.22% for men and women in the third level, respectively. Men in Group 3 with normal BMI and AO increase the risk for type 2 diabetes, with an OR of 4.83 (95% CI: 1.99–11.74) compared with the reference, those in Group 6 and Group 5 with abnormal BMI and P-AO/AO had ORs of 4.05 (95% CI: 2.32–7.08) and 2.98 (95% CI: 1.51–5.87), respectively. For women, subjects of Group 6 with abnormal BMI and AO showed 8.78 times higher risk (OR=8.79, 95%CI: 4.04–19.12) as compared with the reference, followed by Group 3 with normal BMI and AO (OR = 3.30, 95%CI: 1.23–8.86) and Group 5 with abnormal BMI and P-AO (OR = 3.16, 95%CI: 1.21–8.26). A null association was observed between subjects with normal WC but abnormal BMI (Group 4) and risk of type 2 diabetes in either men or women.

## Discussion

In this longitudinal prospective cohort study, we identified associations of combined obesity across BMI- and WC-related single obesity with the risk of type 2 diabetes in Chinese adults aged 18–65, respectively, stratified by gender. We found that men with normal BMI but abdominal obesity were related to higher risk compared with those in other groups, even those with abnormal BMI and abdominal obesity, while women with abnormal BMI and abdominal obesity had highest ORs. Moreover, significant upward trends of ORs were found as BMI and WC increased, and restricted cubic splines model further indicated that the risk of type 2 diabetes had gone up remarkably when BMI was at normal high values (men: BMI ≥ 23.5 kg/m^2^, women: BMI ≥ 22.5 kg/m^2^) and WC at the stage of pre-abdominal obesity (men: WC ≥ 82.0 cm, women: WC ≥ 83.0 cm) in both Chinese men and women.

The refining grouping of obesity based on BMI and WC makes it possible to fully explore the impact of different groups on the risk of type 2 diabetes. We observed that subjects with pre-abdominal or abdominal obesity tended to have higher T2DM risk, regardless of the status of BMI. Several studies reported favorable evidence that stronger association of WC reflecting abdominal obesity with incident type 2 diabetes than for BMI and weight (25, 26). Gulati et al. also found that the pattern of fat deposition in the abdomen was contributory to insulin resistance and diabetes, and it indicated that abdominal obesity could further trigger metabolic abnormalities in individuals (27). Chen et al. in a cohort study of Chinese found that metabolically abnormal but normal-weight individuals had a higher risk of type 2 diabetes than other groups (28), to some extent, the results may be equipped to explain why the individuals with abdominal obesity but the normal weight had a higher risk in our study.

Of note, after stratifying by gender, our study found that women with abnormal BMI and abdominal obesity had highest OR than other groups, in other words, the effect of BMI on the risk of type 2 diabetes in women also had important implications that should not be ignored. Although a meta-analysis reported that abdominal obesity may be a more serious risk factor for diabetes development in women (29), another study cross-sectionally suggested that WC is more closely related to diabetes than BMI, especially in women (30). Moreover, many cohort studies found that women with diabetes had a greatly higher risk of diabetes-related cardiovascular complications compared with men (31). Different body compositions that men had higher lean mass and women had more fat mass for a given BMI (32), sex hormones which were closely related to endocrine environment and energy metabolism, and diversities in biological and psychosocial factors were possibly responsible for gender-specific diabetes risk (33).

In our study, possible exposure–effect relationships between BMI/WC and the risk of type 2 diabetes were also investigated. Results showed that the subjects with normal high values of BMI (men > 23.5 kg/m^2^, women >22.5 kg/m^2^) had significantly increased risk. There has been increasing evidence that BMI cut-offs to prevent obesity-related complications like type 2 diabetes were ethnicity-specific (34, 35), such as a high incident among Chinese populations with a lower BMI (22.2 kg/m^2^, 95% CI: 22.0–22.4) than in White populations (25.0 kg/m^2^) (13), a nationwide survey in Bangladesh also showed that Asian adults with moderately increased BMI (23.0–24.9 kg/m^2^) had increased PR for type 2 diabetes compared with the reference BMI group (18.5–22.9 kg/m^2^) (36). The interactions between gene variants of TCF7L2, GC, and CYP2R1 and BMI or WC may explain the mechanisms underlying the increased risk in the Chinese or Asian populations (37, 38). Similar to BMI, our study suggested that it was associated with higher ORs of developing future diabetes when WC at the stage of pre-abdominal obesity in adults.

In the 10th edition of IDF Diabetes Atlas, it is noteworthy that China has the largest number of diabetes and the highest annual diabetes-related mortality in 2021, and is expected to remain so in 2045 (1). Several studies suggested that trends of type 2 diabetes risk were closely related to obesity and its duration (39), it is worrisome that the prevalence of overweight, obesity, and abdominal obesity increased markedly among Chinese adults over the past two decades (40). Therefore, various factors linked with obesity partly predispose the prevalence and risk to increase in Chinses population, such as sedentary lifestyles, reduced physical activities, prolonged use of electronic devices, sleep quality, convenient transportation and increasing urbanization, energy-dense takeout food and ultra-processed food, eating competence and dietary habits (41). Combined with the results of our study, future research efforts should adopt a comprehensive approach to find sustainable and effective solutions in the reduction of body mass index and modification of body fat distribution, so that we could improve health disparities.

The strengths of our study include reclassifying combined obesity across BMI- and WC-related single groups to fully explore the impact of obesity profiles on the risk of type 2 diabetes in Chinses population, using long-term repeated, subsequent follow-up data, and comprehensive adjustment for potential confounders. Moreover, restricted cubic spline is used to further identify the trends of odds ratios (ORs) under the continuous changes in BMI and WC. And given its many advantages, multilevel mixed-effect modeling is competent to provide a more precise effect estimate instead of traditional regression analyses (42). However, several limitations of our study deserve discussion. First, the lack of a 75-gram oral glucose tolerance test (OGTT) in the survey made it impossible to identify the subjects with impaired glucose tolerance (IGT) and impaired fasting glucose (IFG) as prediabetes. Given the main outcome of type 2 diabetes, the magnitude of obesity, BMI, and WC might be underestimated in this study. Second, although many confounding factors were adjusted, we still cannot exclude the possibility of residual confounding given the nature of observational studies. Third, strict inclusion criteria for the study subjects might reduce the representativeness and generalizability of the findings.

## Conclusion

Briefly, we found that subjects with pre-abdominal or abdominal obesity tended to have higher risk of type 2 diabetes, regardless of the status of BMI, suggesting that WC modification should be given more attention in promoting healthcare. Besides, it was associated with higher risk when BMI at normal high values and WC at the stage of pre-abdominal obesity in Chinese adults. Future studies are warranted to optimize the early screening criteria of BMI and WC based on type 2 diabetes risk in Chinses adults and to promote relevant prevention, early diagnosis, and treatment.

## Data availability statement

The original contributions presented in the study are included in the article/Supplementary material, further inquiries can be directed to the corresponding authors.

## Ethics statement

The studies involving human participants were reviewed and approved by Institutional Review Committees of the University of North Carolina at Chapel Hill and the Chinese Center for Disease Control and Prevention. The patients/participants provided their written informed consent to participate in this study.

## Author contributions

SZ, WL, XJ, and JZ contributed to the study concept and design. SZ performed the statistical analysis and drafted the manuscript. HJ, WL, and LW organized all data. HW and BZ provided statistical expertise. ZW and GD attested that all listed authors meet authorship criteria and that no others meeting the criteria have been omitted, the guarantors of this work and, as such, had full access to all the data in the study and take responsibility for the integrity of the data and accuracy of the data analysis. All authors participated in the interpretation of the results and critical revision of the manuscript.

## Funding

This research was supported by grants from the National Key Research and Development Plan of China (No. 2020YFC2006300), the National Institutes of Health (NIH) (R01-HD30880, DK056350, R24 HD050924, and R01-HD38700), the NIH Fogarty International Center (5D43TW007709 and 5D43TW009077), the Carolina Population Center (5R24 HD050924), the University of North Carolina at Chapel Hill, the National Financial Projects of Public Health Emergency Project-Nutrition Health and Healthy Diet Campaign (No. 131031107000210002), and the Abbott Special Research Fund for food nutrition and safety, Chinese Institute of Food Science and Technology (No. CAJJ-001). The funders had no role in the design of the study; in the collection, analyses, or interpretation of data; in the writing of the manuscript, or in the decision to publish the results.

## Conflict of interest

The authors declare that the research was conducted in the absence of any commercial or financial relationships that could be construed as a potential conflict of interest.

## Publisher's note

All claims expressed in this article are solely those of the authors and do not necessarily represent those of their affiliated organizations, or those of the publisher, the editors and the reviewers. Any product that may be evaluated in this article, or claim that may be made by its manufacturer, is not guaranteed or endorsed by the publisher.
