# Supplementary material for: Association of obesity profiles with type 2 diabetes in Chinese adults: Findings from the China health and nutrition survey
Source: Front Nutr. 2022 Sep 13;9:922824. doi: 10.3389/fnut.2022.922824 (PMC9513418; doi:10.3389/fnut.2022.922824)
Supplement: Supplementary file 1 [file Table_1.DOCX]

| Table S1 Chinese Diabetes Risk Score (BMI,WC) | |
| --- | --- |
| Indicators | Score |
| BMI (kg/m^2^) |  |
| <22.0 | 0 |
| 22.0-23.9 | 1 |
| 24.0-29.9 | 3 |
| ≥30.0 | 5 |
| Waist circumference (cm) |  |
| Men: <75; Women: <70 | 0 |
| Men: 75.0-79.9; Women: 70.0-74.9 | 3 |
| Men: 80.0-84.9; Women:75.0-79.9 | 5 |
| Men: 85.0-89.9; Women: 80.0-84.9 | 7 |
| Men: 90.0-94.9; Women: 85.0-89.9 | 8 |
| Men: ≥95; Women: ≥90 | 10 |
